# Supplementary material for: A Genomic Evaluation of Six Selected Inbred Lines of the Naturalized Plants of Milk Thistle (Silybum marianum L. Gaertn.) in Korea
Source: Plants (Basel). 2023 Jul 20;12(14):2702. doi: 10.3390/plants12142702 (PMC10384142; doi:10.3390/plants12142702)
Supplement: Supplementary file 1 [file plants-12-02702-s001.zip › Supplementary_20230720.pdf]

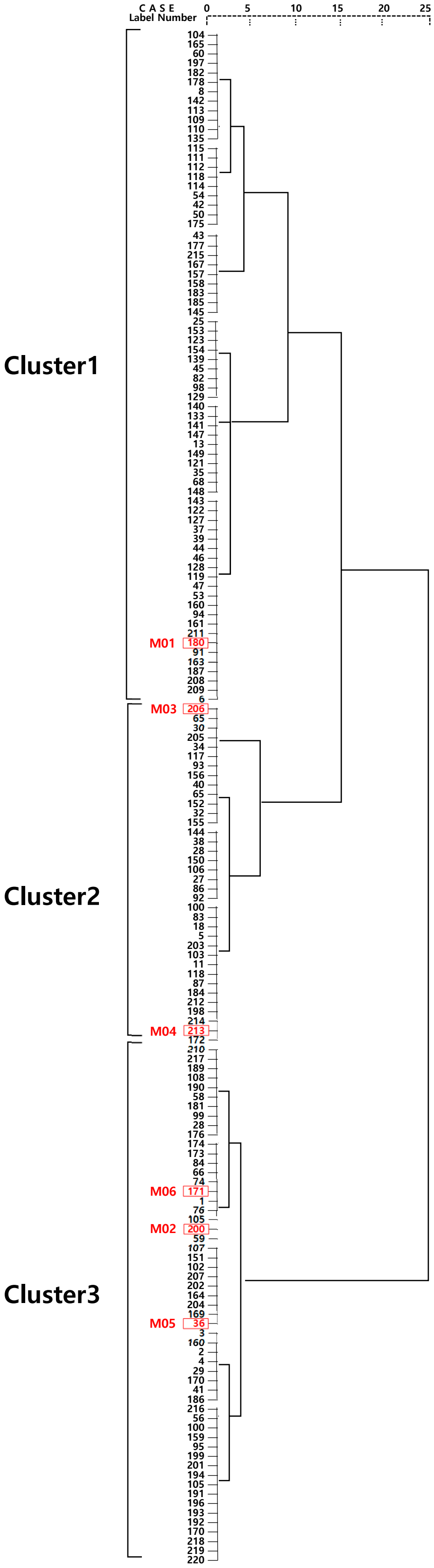

Figure S1. Hierarchical cluster analysis based on agronomical traits with dendrogram visualization by Ward’s method using SPSS.

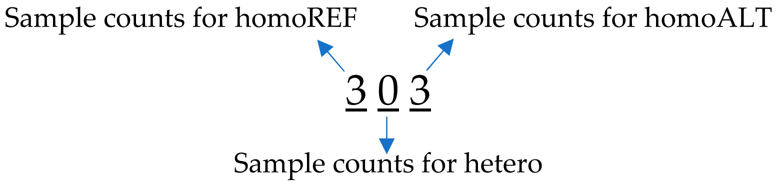

<Example>

Contig name	Position	Contig length	REF.	ALT.	M01	M02	M03	M04	M05	M06	Group	Length Difference
LMWD01002266.1	1251	24953	T	TTGAAGTTATATGAAAAATAT GGCATGATTTGCTTGGGGTC TAAGTTGAAGTTGAGATTGA AGAAAATTAGAAG	1	100	1	100	1	100	303	-73
LMWD01004760.1	5204	21997	G	GTAACACTTACCCGGATAAGA TTTGA	100	1	1	100	1	100	303	-25
LMWD01005723.1	933	7867	CATCTTTTATGTTCAATTTA	C	100	1	100	1	100	1	303	19
LMWD01006025.1	4615	13411	GAGGCAAGCTTCTTCCAAT	G	100	1	1	100	1	100	303	18

Figure S2. An explanation of the modified allele frequency used for indel classification. Sample genotypes were grouped by modified allele frequency which is represented by a polymorphic type of a three-digit number. The first number of three digits represents the number of accessions with homozygotes for the reference allele among 6 accessions, the second digit represents the number of accessions with heterozygote allele, and the third digit represents the number of accessions with homozygotes for the alternative allele, respectively.

Table S1. Correlations between six traits in the 220 milk thistle accessions. Six agronomical traits were evaluated: plant height (PH, cm), seed weight (SW, g), number of flower heads (FHN), SW/FHN, spine length (SL, mm), and plant color at harvest (PC). According to color charts, PC was estimated in three types: green (1), brown (2), and green-brown (3). **$p < 0.01$; *$p < 0.05$.

	PH	SW	FHN	SW/FHN	SL
SW	0.750**				
FHN	0.738**	0.886**			
SW/FHN	0.572**	0.782**	0.499**		
SL	-0.264**	-0.242**	-0.105	-0.340**	
PC	0.433**	0.259**	0.223**	0.218**	-0.259**

Table S2. Phenotypic variation of the six traits in six milk thistle accessions measured in 2019-2021. Means separated within columns followed by different letters were significantly different based on Duncan's test ($P \leq 0.05$).

Sample	Accession	Origin	Plant height (cm)	Branch number	Involucre diameter (mm)	Flower head number	100-achene weight (g)	Spine length (mm)
M01	932014	Canada	111.6±9.6 abc	19.6±0.7 ab	43.3±1.3 b	34.4±2.6 ab	2.1±0.1 d	9.6±1.8 c
M02	932037	Germany	96.3±2.2 c	16.4±1.1 b	46.7±1.4 b	24.2±1.1 b	2.6±0.0 b	15.6±1.3 b
M03	932044	North Korea	95.3±7.1 c	17.2±1.7 b	47.1±1.3 b	31.0±3.1 ab	2.1±0.0 d	10.0±0.8 c
M04	932053	Moldova	124.3±1.9 a	18.8±1.8 ab	55.8±1.6 a	31.4±3.3 ab	2.3±0.1 c	8.0±1.0 cd
M05	912036	Unknown (South Korea)	120.3±4.3 ab	17.4±1.0 b	48.3±2.2 b	39.8±3.3 a	2.8±0.0 a	22.2±1.0 a
M06	912171	Unknown (South Korea)	101.0±4.4 bc	23.4±1.7 a	44.3±0.9 b	31.2±3.5 ab	2.4±0.0 c	4.6±0.7 d

Table S3. Sequence read counts by preprocessing raw resequencing data and trimmed read data by fastq in six milk thistle accessions.

Accession	Raw data		Trimmed data		%
	Total reads	Total bases (bp)	Total reads	Total bases (bp)	
M01	110,905,630	16,746,750,130	108,554,670	16,281,646,777	97.22
M02	139,679,408	21,091,590,608	136,302,598	20,455,574,570	96.98
M03	150,318,804	22,698,139,404	146,110,578	21,919,959,444	96.57
M04	123,412,434	18,635,277,534	121,009,526	18,156,138,037	97.42
M05	149,012,860	22,500,941,860	146,000,972	21,906,263,564	97.35
M06	124,801,536	18,845,031,936	122,017,832	18,314,262,111	97.18
